# The Rubber Dam

**Published:** 1869-02

**Authors:** 


### THE RUBBER DAM.

Frequent inquiries are made in reference to the practicability and efficiency of the rubber dam in filling teeth. We can say, from a pretty thorough experience, that no operator can afford to do without it. There are many cases in which we should not know how to proceed without it. When the web is of the right thickness and properly adjusted, it is a perfect protection against the encroachment of the saliva, and secures to the operator a freedom of action in his manipulations that by no other method, as yet used, is attainable. The preparation and adjustment of the rubber upon the teeth is a very important matter ; indeed, the result wholly depends upon this. If all the teeth are standing when the rubber is to be applied, it should be put over from three to four teeth, or a sufficient number to make the way for the manipulation clear. The holes in the rubber should be from one-eighth to one-fourth of an inch apart, and each perfectly round and smooth cut; this is done with punches made for the purpose; of these there should be about three sizes, varying from one to two lines in diameter. There are also variously formed clamps for holding the rubber down in difficult cases. But a method we think better is the application of the silk ligature about the neck of the tooth, after the rubber is put on. Another important point is the drawing down the edge of the rubber beneath the margin of the gum of the teeth upon which the rubber is placed by the silk thread; this requires some experience and skill to secure the best result. Three things well observed, and any one will be highly pleased with the rubber dam: First—Have a good rubber cloth. Second—Prepare it properly, which consists in cutting it into squares of from four to eight inches, and putting the holes in the proper places and have them nicely cut. Third—Make a perfect adjustment of the rubber on the teeth as indicated above. Some fail in its use for want of a proper knowledge of its application; others fail from pure awkwardness. T.

